# Knockdown of cytokeratin 8 overcomes chemoresistance of chordoma cells by aggravating endoplasmic reticulum stress through PERK/eIF2α arm of unfolded protein response and blocking autophagy

**DOI:** 10.1038/s41419-019-2125-9

**Published:** 2019-11-25

**Authors:** Di Wang, Peiran Zhang, Xiaolong Xu, Jianhui Wang, Dong Wang, Pandi Peng, Chao Zheng, Qing-Jun Meng, Liu Yang, Zhuojing Luo

**Affiliations:** 1 Institute of Orthopedic Surgery, Xijing Hospital, Fourth Military Medical University, Xi’an, 710032 China; 2 Department of Biochemistry and Molecular Biology, Fourth Military Medical University, Xi’an, 710032 China; 3 Medical Research Institute, Northwestern Polytechnical University, Xi’an, 710032 China; 4 Wellcome Centre for Cell Matrix Research, Division of Cell Matrix Biology and Regenerative Medicine, School of Biological Sciences, Faculty of Biology, Medicine and Health, Manchester Academic Health Science Centre, University of Manchester, Manchester, UK

**Keywords:** Bone cancer, Cancer therapeutic resistance

## Abstract

Chordoma is a malignant primary osseous spinal tumor with pronounced chemoresistance. However, the mechanisms of how chordoma cells develop chemoresistance are still not fully understood. Cytokeratin 8 (*KRT8*) is a molecular marker of notochordal cells, from which chordoma cells were believed to be originated. In this study, we showed that either doxorubicin or irinotecan promoted *KRT8* expression in both CM319 and UCH1 cell lines, accompanied by an increased unfolded protein response and autophagy activity. Then, siRNA-mediated knockdown of *KRT8* chemosensitized chordoma cells in vitro. Mechanistic studies showed that knockdown of KRT8 followed by chemotherapy aggravated endoplasmic reticulum stress through PERK/eIF2α arm of unfolded protein response and blocked late-stage autophagy. Moreover, suppression of the PERK/eIF2α arm of unfolded protein response using PERK inhibitor GSK2606414 partially rescued the apoptotic chordoma cells but did not reverse the blockage of the autophagy flux. Finally, tumor xenograft model further confirmed the chemosensitizing effects of siKRT8. This study represents the first systematic investigation into the role of *KRT8* in chemoresistance of chordoma and our results highlight a possible strategy of targeting *KRT8* to overcome chordoma chemoresistance.

## Introduction

Chordoma is a malignant primary osseous spinal tumor (POST) with aggressive local expansion and pronounced chemoresistance^1–3^. It can occur anywhere along the spine, from the skull base to the sacrum, and account for about 20% of POSTs. To date, surgery and adjuvant radiotherapy remain the foundation of the treatment^4,5^. Most median survivals reported are about 5 years in duration due to its high recurrence rates after en bloc resection. Moreover, conventional chordoma were badly insensitive to cytotoxic chemotherapy, which was to be the standard treatment option for metastatic sarcoma^6–9^. Thus, understanding the underlying reasons and molecular mechanisms of chemoresistance of chordoma would be of great help to improve the prognosis of chordoma patients.

Cytokeratins are a subfamily of intermediate filament proteins and are characterized by a remarkable biochemical diversity^10^. Keratin, type II cytoskeletal 8, also known as keratin 8, cytokeratin 8, (*KRT8*, *CK8*, *K8*), a well-known epithelial marker protein, is a molecular marker of notochordal cells, from which chordoma cells were believed to be originated^11–13^. Previous studies have revealed that *KRT8* not only contributes to responding mechanical stress, but also has many significant non-mechanical functions such as signal transduction, stem cell differentiation, and cell protection^10,14–22^. Yet, a role of *KRT8* in chemoresistance has not been documented.

Endoplasmic reticulum (ER), a network of membranous tubules within the cytoplasm of all eukaryotic cell, plays a pivotal role in protein folding, lipid biosynthesis, calcium signaling, and drug detoxification. The accumulation or aggregation of unfolded/misfolded proteins inside the ER induces a cellular condition known as the ER stress and then triggers a set of intracellular signaling pathways collectively referred to as the unfolded protein response (UPR), to transcriptionally and translationally improve ER protein-folding capacity. Three classical arms of UPR are regulated by three ER membrane-embedded sensors: (1) double-stranded RNA-activated protein kinase-like ER kinase (PERK), (2) inositol-requiring enzyme 1 (IRE1), and (3) activating transcription factor 6 (ATF6)^23–26^. Many drug-resistant tumor cells can utilize diverse strategies that enable them to survive the chemotherapy^27^. Drugs disturbing the protein-folding capacity of the ER can provoke ER stress and subsequently induce UPR, endowing malignant cells with greater tumorigenic, metastatic, and drug-resistant capacity^28–30^.

Macroautophagy (hereafter autophagy) serves as an evolutionarily conserved catabolic and quality-control pathway across all eukaryotes^31,32^. The formation of the phagophore, the initial sequestering compartment, which expands into an autophagosome, marks the initiation of the autophagy^33^. Then, autophagosome fuses with lysosomes followed by degradation of the contents, allowing complete flux through the autophagy pathway. In general, autophagy promotes cell survival in response to starvation or other types of cellular stress. Enhanced autophagic responses can support cancer cell survival, proliferation, and growth in adverse microenvironmental conditions, such as the presence of chemotherapy, thereby contributing to drug resistance^34–37^. Unfortunately, the mechanisms of how chordoma cells develop chemoresistance are complicated and still remain elusive.

In the present study, we found the expression of *KRT8* was upregulated in two chordoma cell lines, CM319 and UCH1, after the treatment with doxorubicin (Doxo) or irinotecan (Irino). Therefore, we hypothesized that *KRT8* plays a potential role in chemoresistance of chordoma cells. We then used small interfering (siRNA) to knock down the *KRT8* expression in chordoma cells followed by chemotherapy both in vitro and in vivo, and the results showed that knockdown of *KRT8* overcomes chemoresistance of the chordoma cells through aggravating ER stress, through the PERK/eIF2α arm of UPR and thereby blocking autophagy. The data from this study are the first to provide compelling evidence that upregulation of *KRT8* is one of the mechanism responsible for the chemoresistance of chordoma cells and provided a potential therapeutic approach to overcome chemoresistance of chordoma cells.

## Results

### Doxorubicin or irinotecan significantly promoted *KRT8* expression in chordoma cells in vitro

**Fig. 1:**
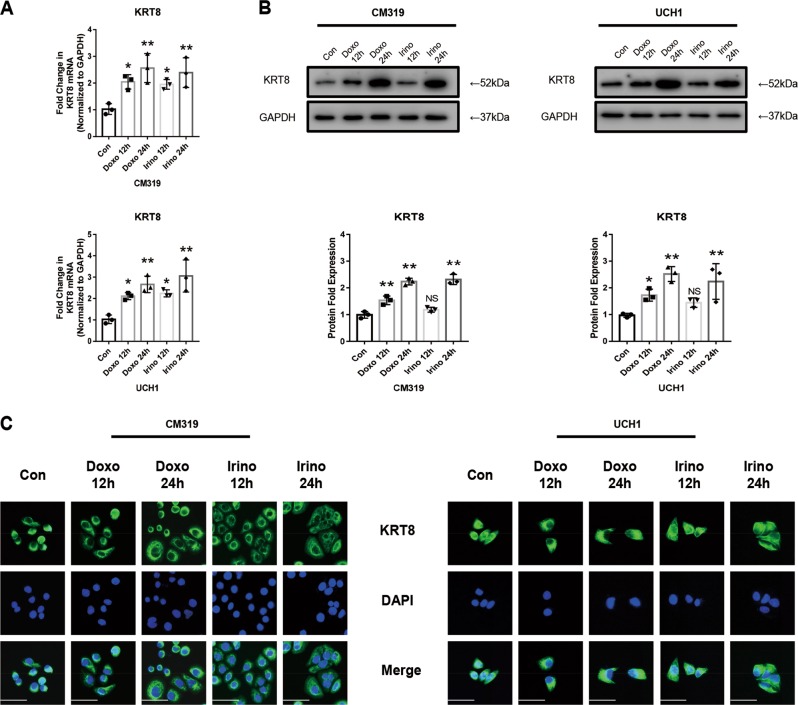
Doxorubicin or irinotecan significantly promoted *KRT8* expression in chordoma cells in vitro. Chordoma cell line CM319 and UCH1 were being treated with doxorubicin (0.5 μM) or irinotecan (50 μM) for 12 h or 24 h. **a** *KRT8* mRNA level was evaluated by qRT-PCR. **b** Western blotting analysis and quantification of KRT8 protein expression (normalized to GAPDH expression). **c** Representative images of immunofluorescence staining of KRT8 of CM318 and UCH1 cell line (*n* = 3, *p < 0.05 vs. control group, **p < 0.01 vs. control group, NS: not statistically significant vs control group, Con: control group, Doxo: doxorubicin-treated group, Irino: irinotecan-treated group. Scale bar = 50 μm. For all the above-mentioned statistical analyses, significance was determined by one-way ANOVA followed by Dunnett’s multiple comparisons test and the results were shown as mean ± SD).

We first investigated the effect of Doxo (0.5 μM) and Irino (50 μM) on *KRT8* expression of CM319 and UCH1 chordoma cells, and found that chemotherapy significantly promoted the expression of *KRT8* in CM319 and UCH1 cells in a time-dependent manner, as shown by the quantitative reverse-transcriptase PCR (qRT-PCR) analysis (Fig. 1a). In addition, consistent with qRT-PCR results, the *KRT8* expression was significantly increased at 24 h in both CM319 and UCH1 cell lines as shown by the western blotting analysis (Fig. 1b). To further investigate the reorganization of KRT8 after chemotherapy, we used immunocytochemistry analysis and the results showed that the *KRT8* expression was promoted throughout the cell in both CM319 and UCH1 cell lines (Fig. 1c). These data indicated that the *KRT8* expression of chordoma cells was significantly increased after chemotherapy.

### Doxorubicin or irinotecan induced UPR and autophagy in chordoma cells in vitro

**Fig. 2:**
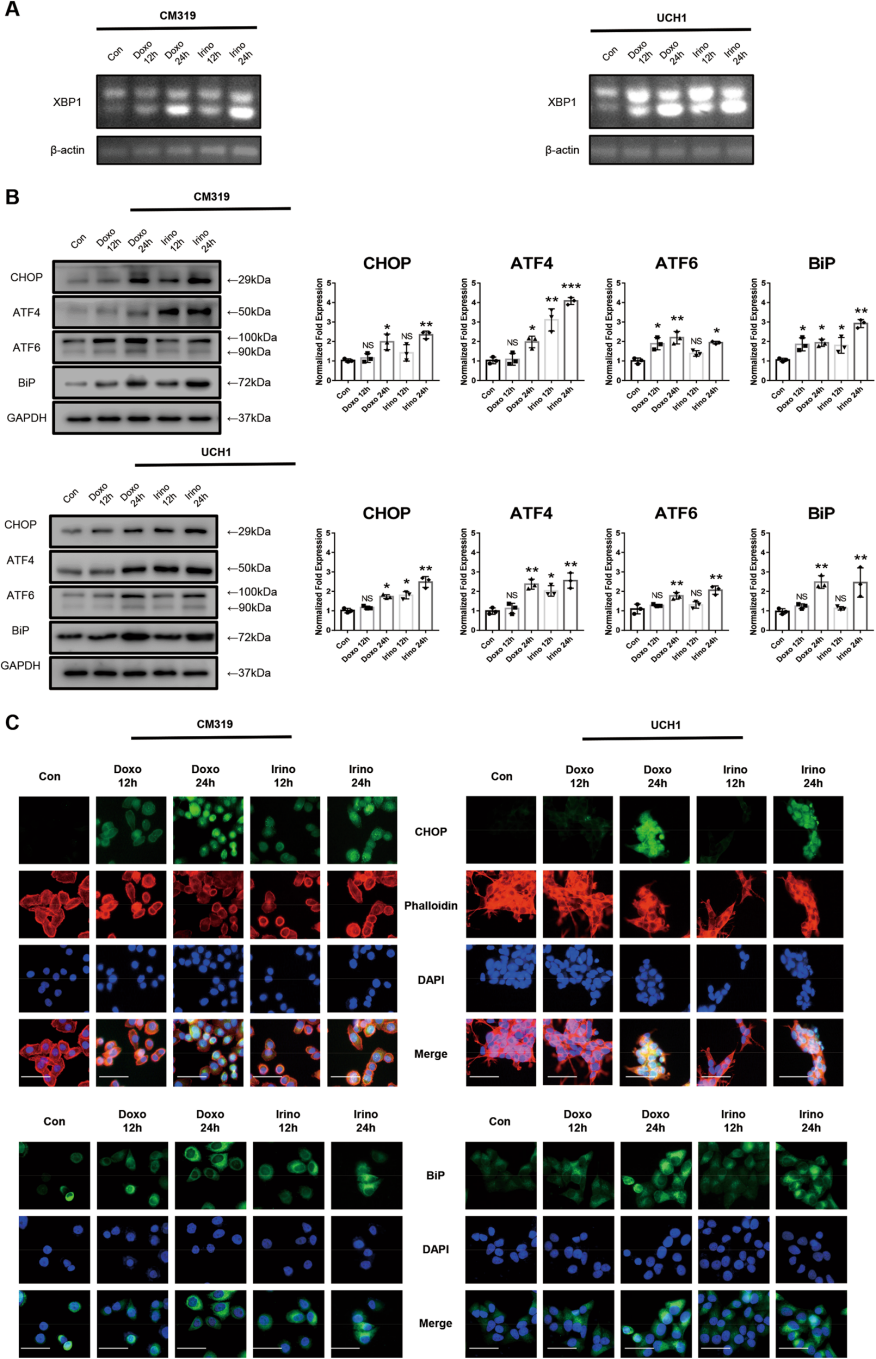
Doxorubicin or irinotecan induced unfolded protein response in chordoma cells in vitro. Chordoma cell lines CM319 and UCH1 were being treated with doxorubicin (0.5 μM) or irinotecan (50 μM) for 12 h or 24 h in vitro. **a** Splicing of *XBP1* mRNA were evaluated by RT-PCR. **b** Western blotting analysis and quantification of CHOP, ATF4, ATF6, and BiP protein expression (normalized to GAPDH expression). **c** Representative images of immunofluorescence staining of CHOP and BiP of CM319 and UCH1 cell line (*n* = 3, *p < 0.05 vs. control group, **p < 0.01 vs. control group, NS: not statistically significant vs. control group, Con: control group, Doxo: doxorubicin-treated group, Irino: irinotecan-treated group. Scale bar = 50 μm. For all the above-mentioned statistical analyses, significance was determined by one-way ANOVA followed by Dunnett’s multiple comparisons test and the results were shown as mean ± SD).

**Fig. 3:**
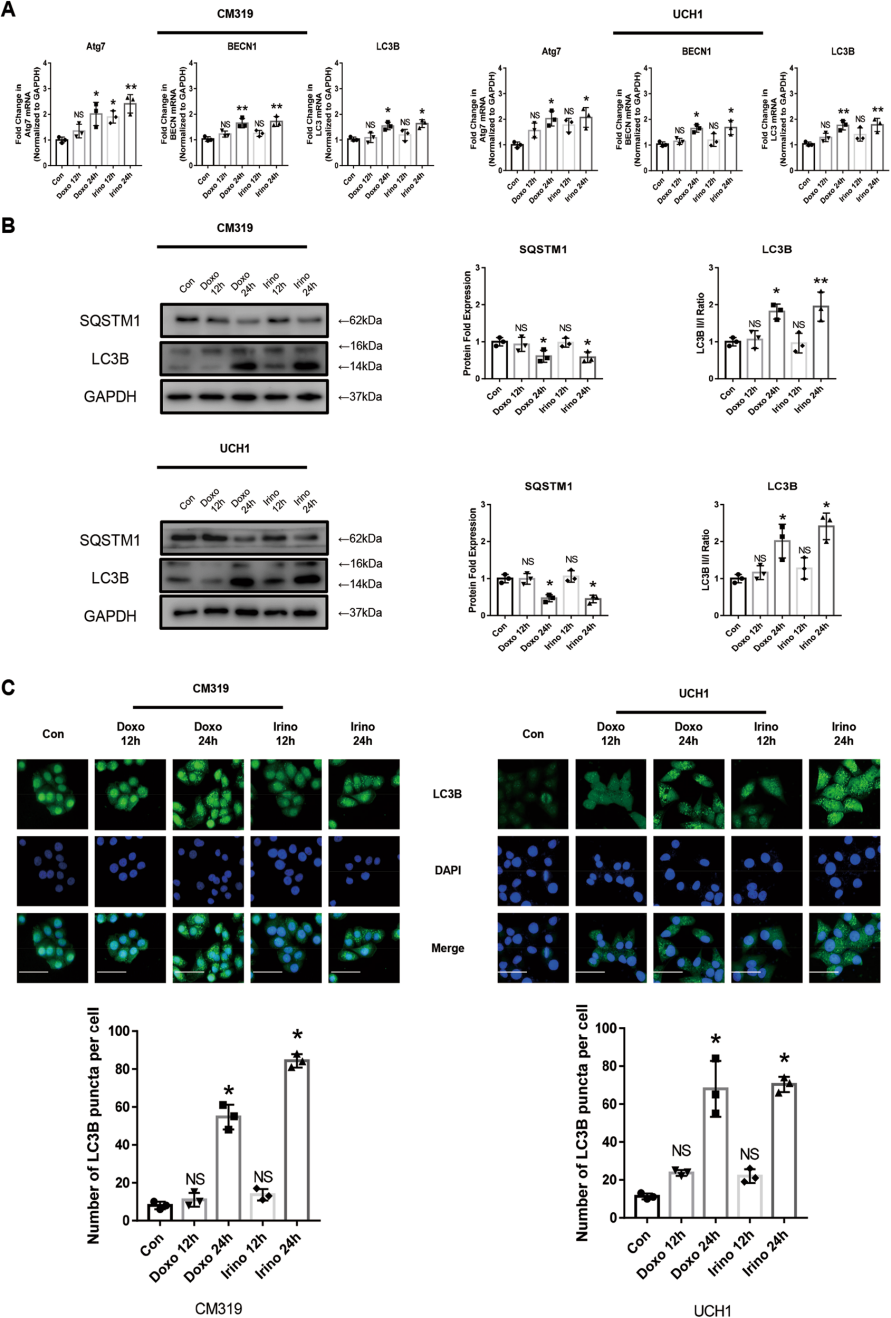
Doxorubicin or irinotecan induced autophagy in chordoma cells in vitro. Chordoma cell line CM319 and UCH1 were being treated with doxorubicin (0.5 μM) or irinotecan (50 μM) for 12 h or 24 h in vitro. **a** *Atg7*, *BECN1*, and *LC3B* mRNA levels were determined by qRT-PCR. **b** Western blotting analysis and quantification of SQSTM1 and LC3B protein expression (normalized to GAPDH expression). **c** Immunofluorescence staining of LC3B and quantification of LC3B puncta per cell of CM319 and UCH1 cell line (*n* = 3, *p < 0.05 vs. control group, **p < 0.01 vs. control group, NS: not statistically significant vs. control group, Con: control group, Doxo: doxorubicin-treated group, Irino: irinotecan-treated group. Scale bar = 50 μm. For all the above-mentioned statistical analyses, significance was determined by one-way ANOVA followed by Dunnett’s multiple comparisons test and the results were shown as mean ± SD).

Human chordoma cells are characterized as large and vacuolized cells, and the notochordal vacuole is thought to be a lysosome-related organelle^9,11,12,38–40^. As lysosome organelles are often related to autophagy activity and ER stress^41,42^, we further explored the UPR and autophagy flux after chemotherapy in chordoma cells. A significant increase of splicing of *XBP1* mRNA was observed after treatment with Doxo (0.5 μM) and Irino (50 μM) for 12 h or 24 h, as shown by RT-PCR, which indicated that the IRE1-α arm of the UPR was activated (Fig. 2a). In addition, the western blotting analysis (Fig. 2b) demonstrated a two-four folds increase of the expression of four main UPR-related proteins, BiP, CHOP (C/EBP Homologous Protein), and ATF4, ATF6 in both CM319 and UCH1 cell lines after treatment with Doxo (0.5 μM) and Irino (50 μM) for 24 h. Furthermore, immunofluorescence analysis also confirmed an elevated ER stress in CM319 and UCH1 cells after treatment with Doxo (0.5 μM) and Irino (50 μM) for 24 h, as indicated by an enhanced expression of CHOP and BiP (Fig. 2c). Besides, we also observed an enhanced autophagy activity from both CM319 and UCH1 cell lines after being treated with Doxo (0.5 μM) and Irino (50 μM) for 12 h or 24 h, indicated by an increased expression of several autophagy-related genes *Atg7*, *BECN1*, and *LC3B*, at the mRNA level (Fig. 3a) and an increased conversion of LC3B I to LC3B II accompanied by a decreased expression of SQSTM1, as determined by the western blotting assay (Fig. 3b). Lastly, the immunofluorescence of LC3B also showed an increased LC3B puncta per cell in both CM319 and UCH1 cell lines after being treated with Doxo (0.5 μM) and Irino (50 μM) for 24 h (Fig. 3c). Collectively, these data showed that chemotherapy induced UPR and autophagy in chordoma cells.

### Knockdown of *KRT8* in chordoma cell increased its’ sensitivity to chemotherapy by promoting its apoptosis in vitro

**Fig. 4:**
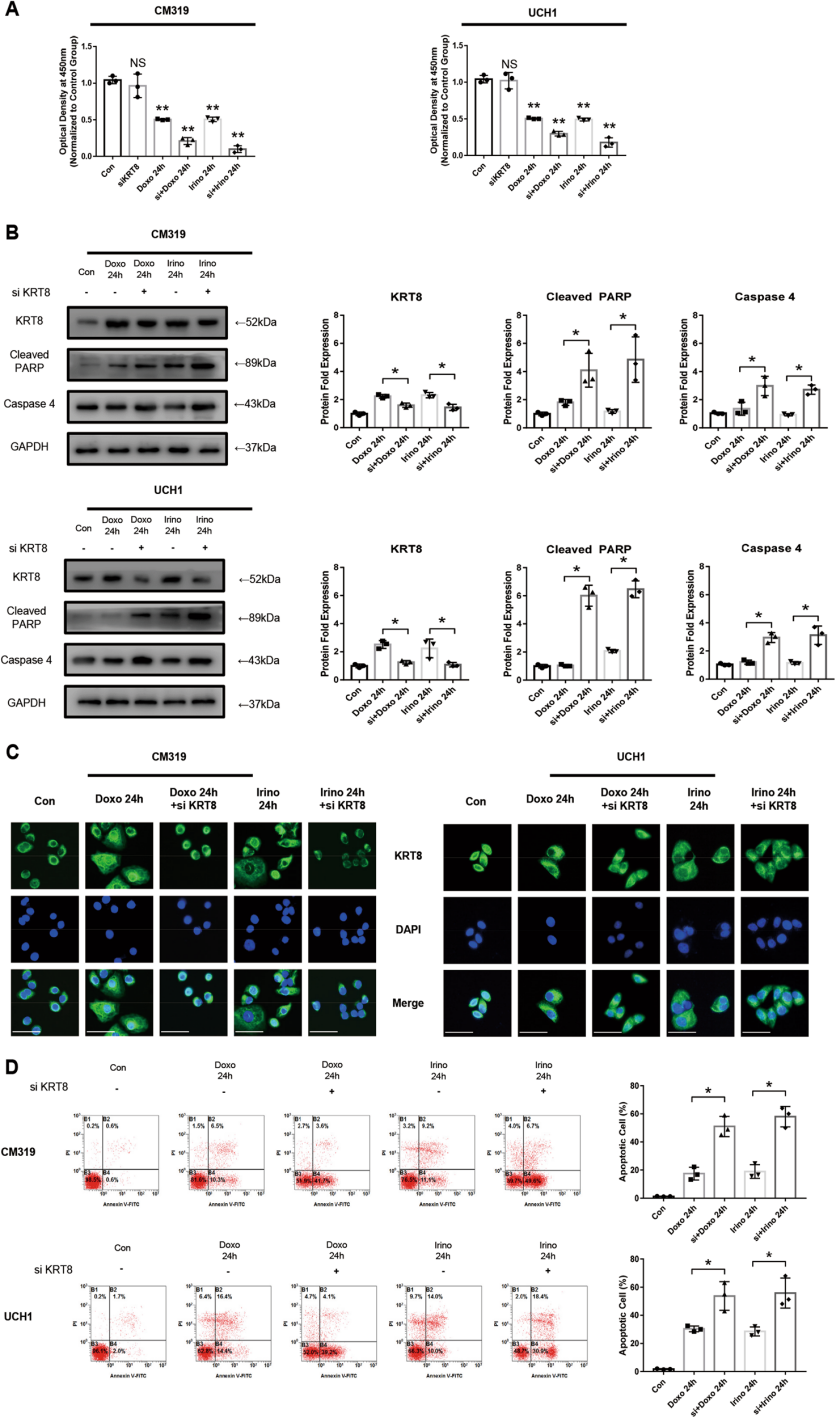
Knockdown of *KRT8* in chordoma cell increased its’ sensitivity to chemotherapy by promoting its apoptosis in vitro. Chordoma cell line CM319 and UCH1 were transfected with siKRT8 followed by treatment of doxorubicin (0.5 μM) or irinotecan (50 μM) for 24 h. **a** Cell viability of chordoma cells were determined by CCK8 assay. **b** Western blotting analysis and quantification of KRT8, cleaved PARP, and Caspase 4 protein expression (normalized to GAPDH expression, quantification data of KRT8 in the “Doxo 24 h” group and “Irino 24h” group were derived from the same data set in Fig. 1b). **c** Immunofluorescence staining of KRT8 of CM319 and UCH1 cell line. **d** Apoptosis of chordoma cells was determined by Annexin V-PE/PI staining measured by flow cytometry (*n* = 3, *p < 0.05 vs. indicated group, **p < 0.01 vs. indicated group, NS: not statistically significant vs. indicated group, Con: control group, Doxo: doxorubicin-treated group, Irino: irinotecan-treated group. Scale bar = 50 μm. For all the above-mentioned statistical analyses, significance was determined by one-way ANOVA followed by Tukey’s multiple comparisons test and the results were shown as mean ± S.D.).

As the expression of *KRT8* of chordoma cells was upregulated after chemotherapy, we hypothesized that *KRT8* plays a potential role in the chemoresistance of chordoma cells. We then used siRNA to knock down the *KRT8* expression of chordoma cells, followed by chemotherapy in vitro. After transfection with siRNA for 24 h, the qRT-PCR and western blotting analysis showed a significant decrease of *KRT8* both at the mRNA and protein level (Fig. S1a, b). Then, we treated the chordoma cells with Doxo (0.5 μM) or Irino (50 μM) for 24 h after transfection with siRNA for 24 h. First, the Cell Counting Kit 8 (CCK8) assay illustrated a significant decrease in cell viability in the “si + doxo” or “si + Irino” group, compared with the “Doxo” or “Irino” group, respectively. However, siKRT8 alone did not affect cell viability on both CM319 and UCH1 cell lines significantly (Fig. 4a). Then, as determined by western blotting analysis and immunofluorescence analysis, the expression of *KRT8* was significantly decreased in “si + doxo” or “si + Irino” group, compared with “Doxo” or “Irino” group, respectively (Fig. 4b, c). In addition, we observed a significant increase in cleaved PARP and Caspase 4 protein expression level, which indicated an increase in ER stress-induced apoptosis (Fig. 4b). Then the Annexin V-PE (Phycoerythrin)/propidium iodide (PI) staining measured by flow cytometry also confirmed a significant increased the apoptosis in “si + doxo” or “si + Irino” group, compared with “Doxo” or “Irino” group, respectively (Fig. 4d). Finally, another set of siRNA (siKRT8–2) was used to rule out the possible “off-target” effect of siRNA (Supplementary Fig. S2). These data illustrated that knockdown of *KRT8* increased its sensitivity to chemotherapy of chordoma cells by promoting its apoptosis.

### Knockdown of *KRT8* followed by chemotherapy promoted apoptosis of chordoma cells through aggregating ER stress through PERK/eIF2α arm of UPR in vitro

**Fig. 5:**
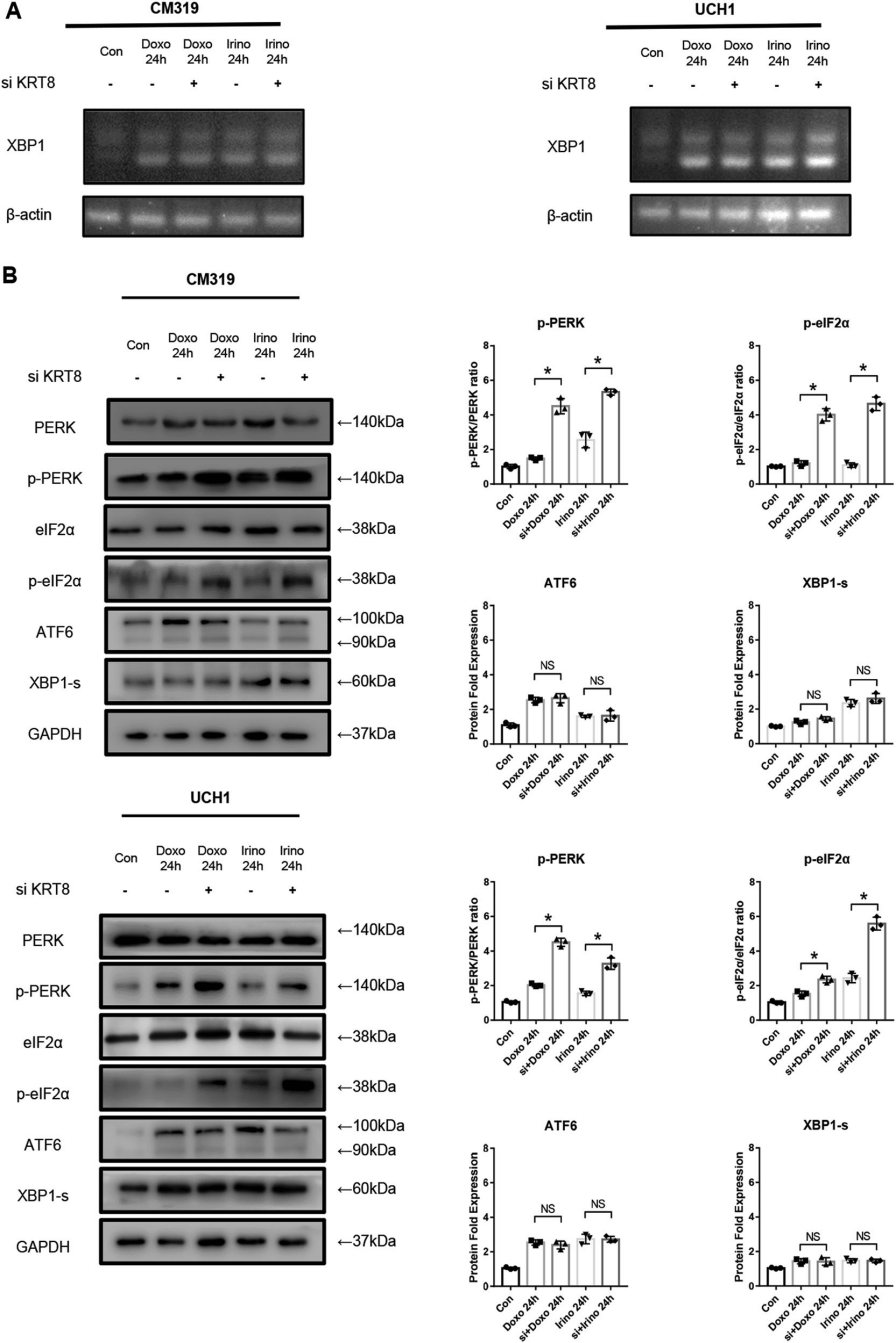
Knockdown of *KRT8* followed by chemotherapy promoted apoptosis of chordoma cells through aggregating ER stress through PERK/eIF2α arm of UPR in vitro. Chordoma cell line CM319 and UCH1 were transfected with siKRT8 followed by treatment of doxorubicin (0.5 μM) or irinotecan (50 μM) for 24 h. **a** Splicing of *XBP1* mRNA were evaluated by qRT-PCR. **b** Western blotting analysis and quantification of PERK, p-PERK, eIF2α, p-eIF2α, ATF6, and XBP1-s protein expression (p-PERK and p-eIF2α were normalized to PERK and eIF2α expression, respectively; ATF6 and XBP1-s were normalized to GAPDH expression; quantification data of ATF6 in “Doxo 24 h” group and “Irino 24 h” group was derived from the same data set in Fig. 2b) (*n* = 3, *p < 0.05 vs. indicated group, **p < 0.01 vs. indicated group, NS: not statistically significant vs. indicated group, Con: control group, Doxo: doxorubicin-treated group, Irino: irinotecan-treated group. For all the above-mentioned statistical analyses, significance was determined by one-way ANOVA followed by Tukey’s multiple comparisons test and the results were shown as mean ± SD).

Disturbance of cellular homeostasis, which leads to accumulation of unfolded/misfolded protein inside the ER, induces a cellular condition known as the ER stress and then triggers a set of intracellular signaling pathways collectively referred to as the UPR, to transcriptionally and translationally cope with the aberrant proteins^25,43,44^. As we have demonstrated that chemotherapy induced UPR in chordoma cells, we further investigated the activation of UPR in cases where *KRT8* was knocked down, followed by chemotherapy. After transfection with siRNA for 24 h, we treated the chordoma cells with Doxo (0.5 μM) or Irino (50 μM) for 24 h. The RT-PCR showed that there were no significant changes in splicing of *XBP1* mRNA level in the “si + doxo” or “si + Irino” group, compared with the “Doxo” or “Irino” group, respectively (Fig. 5a). Consistent with RT-PCR results, there were no significant changes in XBP1-s protein expression level, as determined by western blotting analysis in these groups (Fig. 5b). Similar to that, the ATF6 protein expression level also showed no significant changes in these groups (Fig. 5b). However, as determined by western blotting analysis, the phosphorylation of PERK and eIF2α, the two major components of PERK/eIF2α arm of the UPR, were strikingly increased in “si + doxo” or “si + Irino” group, compared with “Doxo” or “Irino” group, respectively (Fig. 5b). However, no significant changes in protein expression level of BiP, CHOP in “siKRT8” group, compared with “siNC” group, were observed (Supplementary Fig. S1b). These data clearly showed that knockdown of *KRT8* followed by chemotherapy significantly aggregate ER stress through the PERK/eIF2α arm of UPR.

### Knockdown of *KRT8* followed by chemotherapy blocked the late stage of autophagy in vitro

**Fig. 6:**
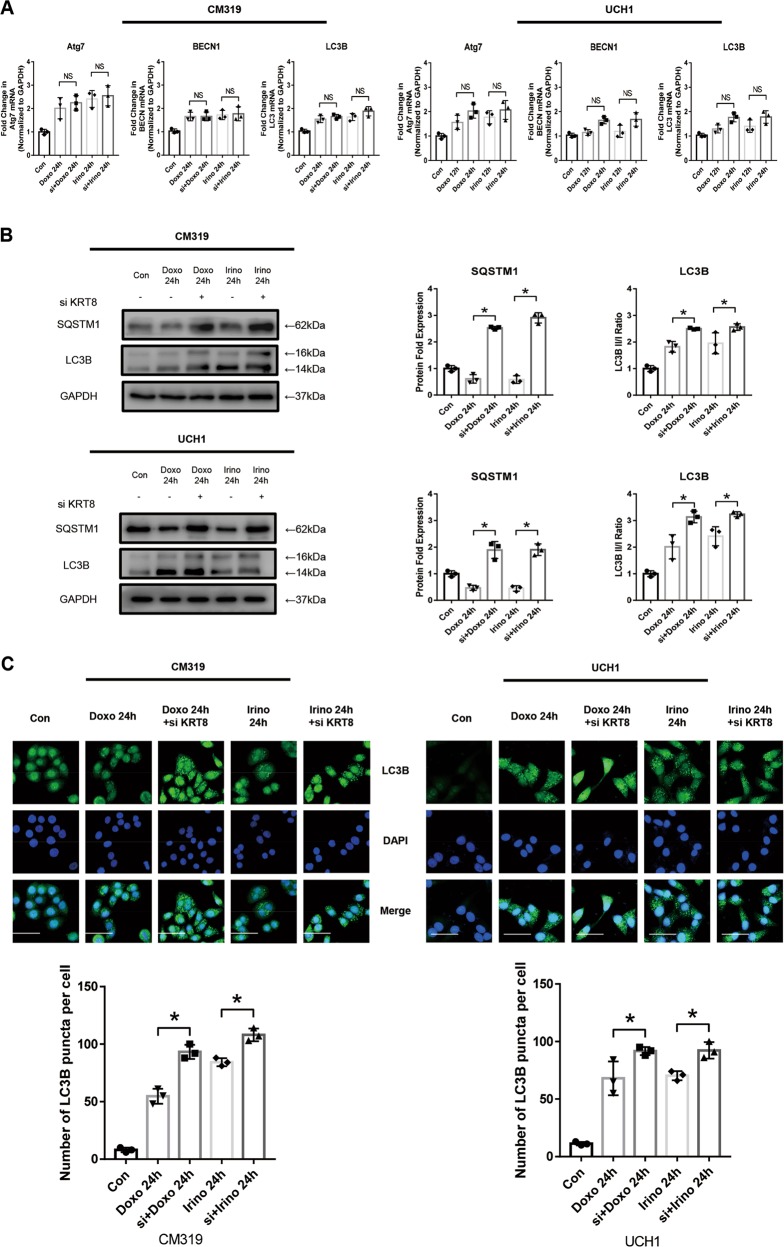
Knockdown of *KRT8* followed by chemotherapy blocked the late stage of autophagy in vitro. Chordoma cell line CM319 and UCH1 were transfected with siKRT8 followed by treatment of doxorubicin (0.5 μM) or irinotecan (50 μM) for 24 h. **a** *Atg7*, *BECN1*, and *LC3B* mRNA level were determined by qRT-PCR. **b** Western blotting analysis and quantification of SQSTM1 and LC3B protein expression (normalized to GAPDH expression, quantification data of LC3B and SQSTM1 in “Doxo 24 h” group and “Irino 24 h” group was derived from the same data set in Fig. 3b). **c** Immunofluorescence staining of LC3B and quantification of LC3B puncta per cell of CM319 and UCH1 cell line (quantification data of LC3B puncta in “Doxo 24 h” group and “Irino 24 h” group was derived from the same data set in Fig. 3c) (*n* = 3, *p < 0.05 vs. indicated group, **p < 0.01 vs. indicated group, NS: not statistically significant vs. indicated group, Con: control group, Doxo: doxorubicin-treated group, Irino: irinotecan-treated group. Scale bar = 50 μm. For all the above-mentioned statistical analyses, significance was determined by one-way ANOVA followed by Tukey’s multiple comparisons test and the results were shown as mean ± SD).

Recently, ample studies showed that enhanced autophagic responses can support cancer cell survival, proliferation, and growth in adverse microenvironmental conditions, such as the presence of chemotherapy, and therefore contributes to drug resistance^32,36^. As we have showed that chemotherapy could promote autophagy activity in chordoma cells, we further investigated the autophagy flux in cases where *KRT8* was knocked down followed by chemotherapy. After transfected with siRNA for 24 h, we treated the chordoma cells with Doxo (0.5 μM) or Irino (50 μM) for 24 h. As determined by qRT-PCR, there were no significant changes in *Atg7*, *BECN1*, and *LC3B* mRNA level in “si + doxo” or “si + Irino” group, compared with “Doxo” or “Irino” group, respectively (Fig. 6a). However, the western blotting analysis showed a significant higher LC3B II/I ratio in these groups (Fig. 6b). Consistent with western blotting results, the immunofluorescence of LC3B also showed a significant increase of LC3B puncta per cell in these groups (Fig. 6c). Moreover, as determined by western blotting, the SQSTM1 expression level was significantly increased in “si + doxo” or “si + Irino” group, compared with “Doxo” or “Irino” group, respectively (Fig. 6b). However, no significant changes in protein expression level of LC3B, SQSTM1 in “siKRT8” group, compared with “siNC” group were observed (Fig. S1b). As LC3B I to LC3B II conversion occurred at an early stage of autophagy and SQSTM1 degradation occurred at a late stage of autophagy, the knockdown of *KRT8* followed by chemotherapy induced a blockage of late-stage autophagy.

### PERK inhibitor GSK2606414 decreased UPR activation and partially abolished chemosensitizing effect of siKRT8 but did not reverse the blockage of autophagy flux in vitro

**Fig. 7:**
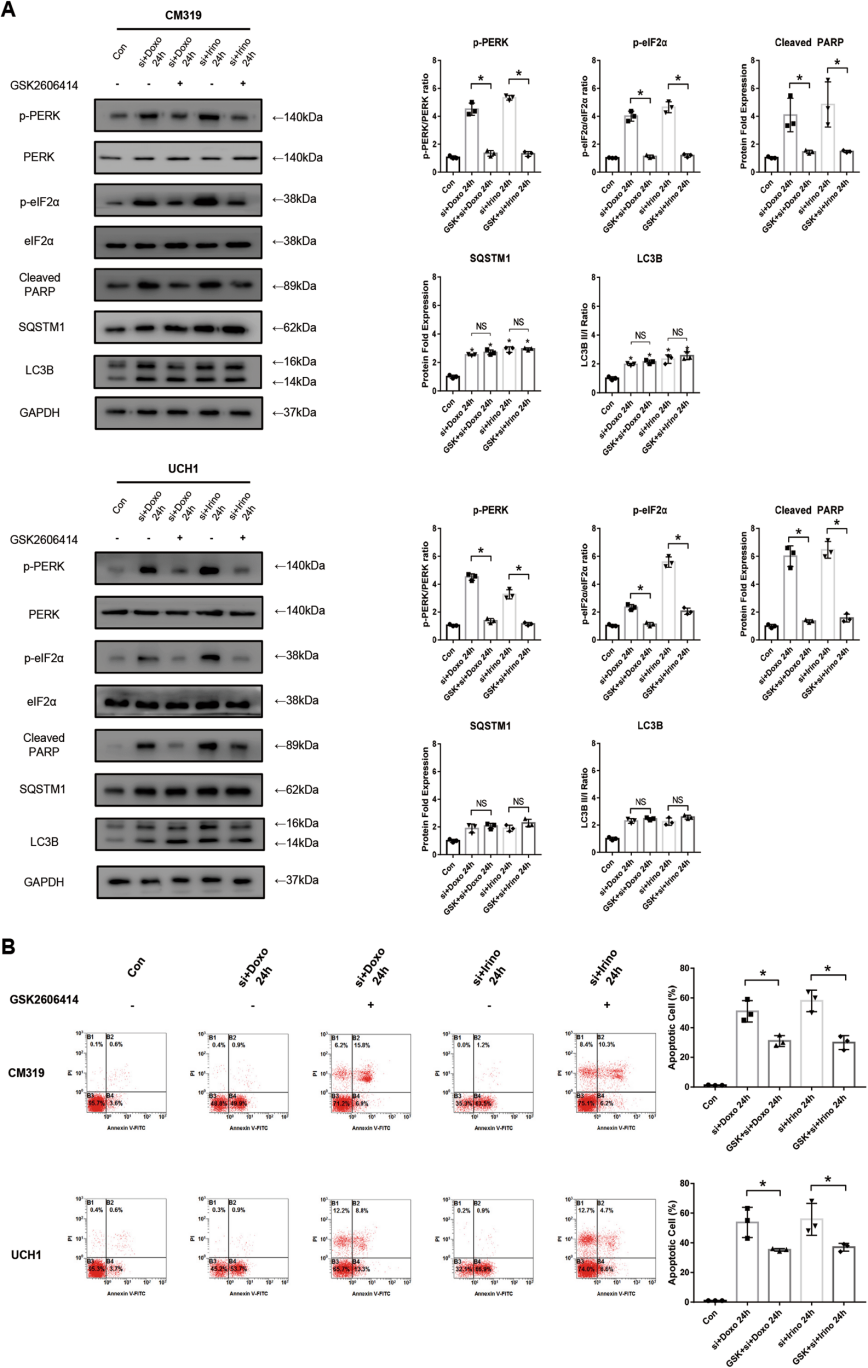
PERK inhibitor GSK2606414 decreased UPR activation and partially abolished chemosensitizing effect of siKRT8 but did not reverse the blockage of autophagy flux in vitro. Chordoma cell line CM319 and UCH1 were transfected with siKRT8 followed by treatment of doxorubicin (0.5 μM), irinotecan (50 μM), and GSK2606414 (2 μM) for 24 h. **a** Western blotting analysis and quantification of p-PERK, p-eIF2α, Cleaved PARP, SQSTM1, and LC3B protein expression (p-PERK and p-eIF2α were normalized to PERK and eIF2α expression, respectively; Cleaved PARP, LC3B, and SQSTM1 were normalized to GAPDH expression; quantification data of p-PERK, p-eIF2α, Cleaved PARP, SQSTM1, and LC3B in “si + Doxo 24 h” group and “si + Irino 24 h” group were derived from the same data set in Figs. 4b and 5b). **b** Apoptosis of chordoma cells was determined by Annexin V-PE/PI staining measured by flow cytometry (quantification data of apoptosis in “si + Doxo 24 h” group and “si + Irino 24 h” group were derived from the same data set in Fig. 4d) (*n* = 3, *p < 0.05 vs. the indicated group, **p < 0.01 vs. the indicated group, NS: not statistically significant vs. indicated group, Con: control group, Doxo: doxorubicin-treated group, Irino: irinotecan-treated group. For all the above-mentioned statistical analyses, significance was determined by one-way ANOVA followed by Tukey’s multiple comparisons test and the results were shown as mean ± SD).

To further elucidate the relationship between the elevated UPR and the blockage of autophagy flux in cases where knockdown of *KRT8* was followed by chemotherapy, we used specific PERK inhibitor GSK2606414 (2 μM) to inhibit the activation of PERK arm of UPR after knockdown of *KRT8* and chemotherapy. Unsurprisingly, GSK2606414 abolished the activation of PERK/eIF2α arm of UPR brought by knockdown of *KRT8* followed by chemotherapy, as shown by decreased p-PERK and p-eIF2α expression (Fig. 7a). Also, the inhibition of UPR significantly rescued the apoptosis of chordoma cells, as shown by decreased expression of cleaved PARP (Fig. 7a). Consistent with western blotting results, the Annexin V-PE/PI staining measured by flow cytometry also confirmed a significant decreased apoptosis after treatment of GSK2606414 (Fig. 7b). However, we did not observe a significant change of LC3B II/I ratio and SQSTM1 protein expression after the inhibition of PERK/eIF2α arm of UPR by GSK2606414 (Fig. 7a), which indicated that the blockage of autophagy was independent of the activation of UPR. These data showed that PERK inhibitor GSK2606414 decreased UPR activation and partially abolished chemosensitizing effect but did not reverse the blockage of autophagy flux brought up by the knockdown of *KRT8*.

### Knockdown of *KRT8* increased chemosensitivity of chordoma cells in vivo

**Fig. 8:**
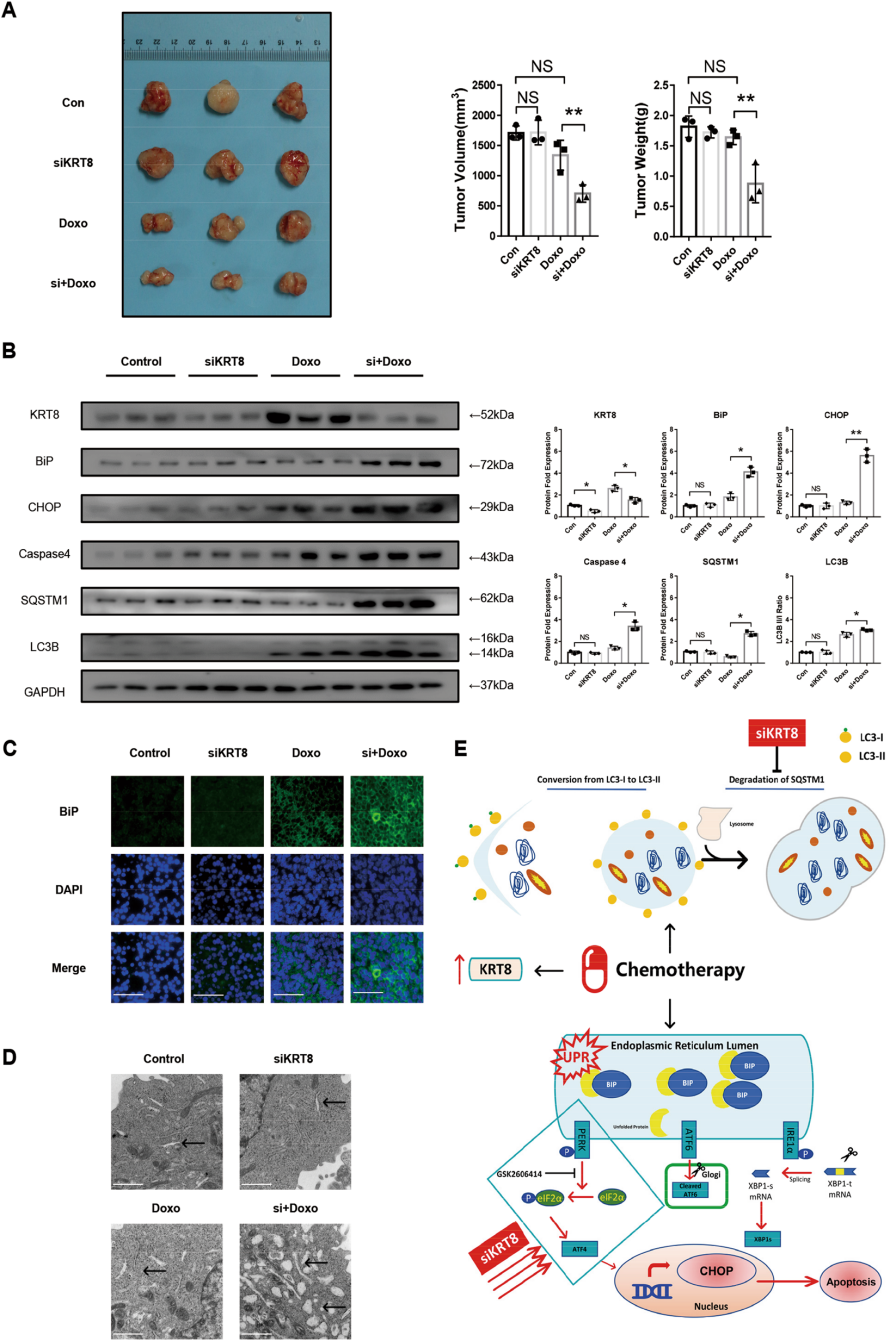
Knockdown of *KRT8* increased chemosensitivity of chordoma cells in vivo. NOD/SCID mice bearing CM319 cell xenografts were divided into four groups: three mice for each group—(1) control group (Con), (2) siKRT8 group (siKRT8), (3) doxorubicin group (Doxo, 2 mg/kg), and (4) siKRT8 and doxorubicin group (si + Doxo 2 mg/kg). **a** siKRT8 combined with doxorubicin exerted a more significant anti-tumor effect as shown by tumor weights and volume. **b** Western blotting analysis and quantification of KRT8, BiP, CHOP, Caspase 4, SQSTM1, and LC3B protein expression of tumor xenograft tissues (normalized to GAPDH expression). **c** Immunofluorescence staining of BiP of tumor xenograft sections (scale bar = 100 μm). **d** Representative TEM image of tumor tissues, ER was evidently distended in si + Doxo-treated groups (arrowhead, scale bar = 1μm). **e** Proposed mechanism of *KRT8* in chordoma drug resistance. *KRT8* of chordoma cells is upregulated after chemotherapy, accompanied by increased autophagy activity and ER stress. Knockdown of *KRT8* sensitizes chordoma cells to chemotherapy by blocking late-stage autophagy and aggravating ER stress through PERK/eIF2α arm of UPR (*n* = 3, *p < 0.05 vs. indicated group, **p < 0.01 vs. indicated group, NS: not statistically significant vs. indicated group, Con: control group, Doxo: doxorubicin-treated group. For all the above-mentioned statistical analyses, significance was determined by one-way ANOVA followed by Tukey’s multiple comparisons test and the results were shown as mean ± SD).

As we have clearly showed that knockdown of *KRT8* overcomes chemoresistance of chordoma cells by promoting its apoptosis in vitro, to further examine the chemosensitizing effect of siKRT8 in vivo we developed a xenograft model using the CM319 cell line. Briefly, CM319 cells were subcutaneously injected into NOD/SCID (Non-obese diabetic/scid gamma) mice. When the mean tumor volume reached 500 mm^3^ (15 days after the injection of CM319 cells), the NOD/SCID mice were randomly assigned to four groups: three mice for each group—control group (Con), siKRT8-treated group (siKRT8), Doxo (2 mg/kg), and siKRT8 and Doxo group (si + Doxo 2 mg/kg). Doxo were intraperitoneally administered every day for 14 days and siRNA was transfected three times weekly. After 30 days from the injection of the CM319 cells, the mice were killed and xenografts were collected for weighing and histology analysis. The results showed that the “si + Doxo” treatment resulted in a significant inhibition on tumor growth compared with the “Doxo” group and control group (Fig. 8a); yet, the “siKRT8” group showed no significant changes in terms of tumor growth (Fig. 8a). Western blotting analysis showed a significant increase in BiP, CHOP, Caspase 4 protein expression in “si + Doxo” group, compared with control group or “si + Doxo” group, which indicated a promotion of ER stress-induced cell death (Fig. 8b). Also, the western blotting analysis showed an increased SQSTM1 protein expression level in the “si + Doxo” group compared with the “Doxo” group, which indicated a blockage of autophagy flux (Fig. 8b). However, the “siKRT8” group showed no significant changes in protein expression of BiP, CHOP, Caspase 4, SQSTM1, and LC3B, compared with the control group (Fig. 8b). Immunofluorescence staining of tumor sections also confirmed an increase in BiP expression in the “si + Doxo” group compared with the “Doxo” group and control group (Fig. 8c). Consistently, transmission electron microscopic analysis of tumor tissue showed aberrant distension of the ER in the “si + Doxo” group compared with the control group and “Doxo” group (Fig. 8d). These data showed that knockdown of *KRT8* increased chemosensitivity of chordoma cells through aggravating ER stress and blocking late-stage autophagy in vivo.

## Discussion

Chordoma is originated from notochord remnants, which were characterized as large vacuolized cells^13,45,46^. Its high insensitivity to chemotherapy and the lack of curative therapy warrant the investigation into new therapeutic approaches^4,6^. Doxo is a classical topoisomerase II inhibitor, which chordoma cells are relatively sensitive to^38,39^. Irino, a topoisomerase I inhibitor, is one of the camptothecin analogs that has been approved for cancer treatment and is the only chemotherapy agent that has finished phase II study on chordoma^4,47^. In this study, we found that either 0.5 μM Doxo or 50 μM Irino induce apoptosis of chordoma cells, after being treated for 24 h. However, a difference of chemoresistance between CM319 and UCH1 cell lines is observed. As determined by Annexin V-PE/PI apoptosis assay, CM319 cell line is more resistant to Doxo than UCH1 cell line; yet, both CM319 and UCH1 cell lines show a similar sensitivity to Irino.

Keratins have a cell-specific expression pattern, which is employed to distinguish different epithelial cell types. *KRT8* expresses in many tissues, including the liver, retina, pancreas, and intestine, executing multiple cellular functions, including mechanical support, stem cell-fate control, and cytoprotective response^10,14,15,17–20,22^. Pekny et al.^48^ reported that keratins were upregulated during or recovering from stress. The study conducted by Ku and Omary^15^ showed that *KRT8* could serve as a “phosphate sponge” and thus protect liver cells from stress-induced apoptosis. In addition, Lähdeniemi et al.^19^ demonstrated that *KRT8* and Notch1 colocalized and interacted with each other in the colonic epithelial cells. *KRT8* could also enhance Notch1 protein levels and activity in a dose-dependent manner and regulate Notch1 signaling activity and differentiation in the epithelium of the large intestine^19^. Also, a previous study conducted by Trisdale et al.^22^ showed an increased FAS expression and a decreased resistance to Fas-activating antibody after knockdown of *KRT8* in granulosa cell tumors. Consistent with their results, we also find *KRT8* plays an important role in chordomas’ apoptosis resistance. However, we do not observe an increased expression of FAS after knockdown of *KRT8* alone or followed by treatment of Doxo or Irino as determined by qRT-PCR, and this may due to the low expression of FAS in chordoma cells (data not shown). The data from this study show that *KRT8* is significantly upregulated after treatment with Doxo and Irino for 24 h. Moreover, immunocytochemistry analysis further demonstrates that *KRT8* expression is promoted throughout the cell in both CM319 and UCH1 cell lines. Then, our data show that siRNA-mediated knockdown of *KRT8* alone do not promote cell apoptosis, nor does it induce UPR and autophagy in chordoma cells. However, after being treated with Doxo or Irino, strikingly increased apoptosis is observed. Collectively, in this study, we show that *KRT8* executes a cytoprotective action during chemotherapy and endows chordoma cells with greater chemoresistance. In addition, knockdown of *KRT8* sensitizes both CM319 and UCH1 cell lines to both Doxo and Irino, which shows that *KRT8* may serve as a universal therapeutic target for chemoresistant chordoma.

The ER fulfills multiple cellular functions. A number of stresses, including nutrient deprivation, high metabolic demand, and chemotherapies, disturbed the protein-folding capacities within the ER, thus triggering an evolutionarily conserved response, collectively referred to as UPR. The UPR is considered as a significant factor in many pathologies, including cancer, liver dysfunction, neurodegenerative diseases, and ischemia^27,49–52^. To date, emerging evidence from many studies have revealed that activation of UPR endows tumor cells with great drug-resistant capacity. In general, the activation of UPR initially favors a cytoprotective response; however, it also paradoxically induces apoptosis when cells fail to adapt to severe ER stress^25,53–55^. In this study, we find that Doxo or Irino induce a moderate UPR in chordoma cells as shown by an increased expression of CHOP, BiP, ATF4, ATF6, and XBP1-s. In addition, a difference of the arms of UPR induced was observed between Doxo and Irino. As shown by western blotting analysis, Doxo preferentially induces ATF6 arm of UPR, whereas Irino preferentially induces ATF4 arm of UPR, in both CM319 and UCH1 cell lines. This discrepancy may be explained by the different pharmacological action of these two drugs (Irino is a topoisomerase I inhibitor, whereas Doxo is a topoisomerase II inhibitor). Then we further investigate the role of *KRT8* during UPR and the data show that siRNA-mediated knockdown of *KRT8* alone does not provoke significant changes in UPR. However, after treatment with Doxo or Irino, a strikingly elevated expressions of CHOP, BiP are observed, which represents an increase in ER stress. Moreover, we also find that knockdown of *KRT8* aggravates ER stress through the PERK/eIF2α arm of UPR, as shown by an increased expression of p-PERK and p-eIF2α expression.

Autophagy can be induced by nutrient deprivation, ER stress, and chemotherapy, functioning as a pro-survival strategy. Ample studies and clinical trials supported autophagy as a potential yet potent therapeutic target for many drug-resistant malignant tumors^34,35,37,56^. The large vacuole of chordoma cells are considered as a lysosome-related organelle and some studies tried to link these large vacuoles to drug-resistant capacity by targeting autophagosome–lysosome fusion using chloroquine, an effective anti-malaria drug. However, the results showed that chloroquine only exerted a weak growth inhibition on chordoma cells and could only serve as a weak chemosensitizer for doxorubicin. Moreover, this chemosensitizing effect was only observed in the MUG-Chor1 cell line but not in the MUG-CC1 cell line; this may be because the autophagy flux of chordoma cells cannot be inhibited within the therapeutic concentration window of chloroquine. Despite this, Kolb‑Lenz et al.^38^ still believed that the huge lysosomal mass of chordoma might in fact represent a drug target, which is worth testing in further studies. The connection between *KRT8* and autophagy has been reported. Kongara et al.^57^ showed that high phosphor (Ser73)-KRT8 levels are inversely correlated with Beclin 1 expression in human breast tumor cells. Furthermore, phosphor (Ser73)-KRT8 was accumulated in metabolically stressed immortalized mammary epithelial cells in a SQSTM1-dependent manner upon autophagy inhibition^57^. In addition, Baek et al.^16^ showed that knockdown of *KRT8* affected the fusion between autophagosomes and lysosomes after being treated with paraquat, and increased expression of *KRT8* following autophagy provides a cytoprotective role in retinal pigment epithelium. Consistently, our data show an elevated autophagy activity within the chordoma cells after chemotherapy. Moreover, knockdown of *KRT8*, followed by treatment with Doxo or Irino, prevents SQSTM1 from degradation as shown by western blotting, which indicates that the late stage of autophagy is blocked. In addition, we further elucidate that the blockage of autophagy flux brought by *KRT8* knockdown is independent of the activation of the PERK/eIF2α arm of UPR, as no significant change in SQSTM1 expression is observed, after treatment of PERK inhibitor GSK2606414. However, despite the fact that the PERK inhibitor GSK2606414 abolishes the elevated UPR and partially rescues chordomas’ apoptosis, the apoptotic ratio is still significantly higher than the “Doxo” or “Irino” group, as determined by two-tailed unpaired Student’s *t*-test (data not shown). A most likely explanation is that knockdown of *KRT8* successfully blocks the late-stage autophagy and therefore chemosensitizes chordoma cells.

To conclude, in the present study, we find that *KRT8* is upregulated after treatment with Doxo and Irino. In addition, upregulated *KRT8* is accompanied by induction of autophagy and UPR. Knockdown *KRT8* chemosensitizes chordoma by strikingly aggravating ER stress through the PERK/eIF2α arm of UPR and blocks the late-stage autophagy. In addition, the blockage of late-stage autophagy is independent of the activation of the PERK/eIF2α arm of UPR. Lastly, a xenograft model using CM319 cell line further confirms that knockdown of *KRT8* increases chemosensitivity of chordoma in vivo. This study represents the first systematic investigation into the role of *KRT8* in chemoresistance of chordoma cells and our results suggest *KRT8* as a promising therapeutic target for chemoresistant chordoma.

## Materials and methods

### Cell culture and reagents

Human chordoma cell line CM319^2,58^ was a gift from Dr. Wen Yan-hua (Department of Orthopedic Oncology, Tang Du Hospital, Xi’an, China) and was maintained at 37 °C under 5% CO_2_ in RPMI 1640 medium (Gibco, USA) supplemented with 10% fetal bovine serum (FBS) (Gibco, USA), penicillin (100 units/ml), and streptomycin (100 μg/ml). Human chordoma cell line UCH1^13,39,40^ was obtained from American Type Culture Collection (#CRL­3217) and was maintained in rat tail type I collagen (BD Biosciences)-coated flasks at 37 °C under 5% CO_2_ in Iscove/RPMI (4:1) medium (Gibco, USA), supplemented with 10% FBS (Gibco, USA), penicillin (100 units/ml), streptomycin (100 μg/), and 1% (v/v) l-glutamine (Gibco, USA). Doxo (#HY-15142A), Irino (#HY-16562), and GSK2606414 (#HY-18072) were purchased from MedChemExpress (Shanghai, China). The final concentration of dimethylsulfoxide is <0.05% (v/v). No mycoplasma was detected in the CM319 and UCH1 cell lines.

### siRNA transfection in vitro

The siRNA duplexes targeting human *KRT8* and negative control (NC) siRNA were designed and synthesized by Genepharma (Suzhou, China). siRNA sequences used in this study were shown in Supplementary Table 1. siRNA transfection was performed using Lipofectamine 2000 Reagent (Invitrogen, USA) according to the manufacturer’s instructions. Briefly, chordoma cells were seeded (2.5 × 10^5^) in a six-well plate, after adhesion, and the cells were incubated with Optic-MEM medium (Thermo Fisher Scientific, USA, #31985062) at 37 °C for 2 h. Cells were then transfected with 100 nM of *KRT8* siRNA or NC siRNA using Lipofectamine 2000 Reagent (Invitrogen, USA) and were incubated at 37 °C for 4–6 h. Finally, cells were washed with phosphate-buffered saline (PBS) and changed the medium with complete culture medium. Cells were collected for qRT-PCR or western blotting, to evaluate the knockdown efficacies 24 h later. In some experiments, knockdown of *KRT8* was followed by treatment with Doxo, Irino, or GSK2606414.

### Cell viability assay

Cell viability was evaluated by CCK8 (Dojindo, Kumamoto, Japan) according to the manufacturer’s instructions. Briefly, CM319 and UCH1 cells were seeded into 96-well plates at a density of 5000/well with 3 replicates. After adhesion, the cells were treated accordingly. Then the original medium was replaced by a mixture of 10 μl CCK8 reagent and 100 μl fresh medium, and were incubated at 37 °C for 4 h. Finally, the optical density of each well was measured by a microplate reader (BioTek, USA) at 450 nm.

### Flow cytometry apoptosis assay

Chordoma cells were seeded in to a six-well plate and treated with indicated chemicals for desired period. After treatment, the chordoma cells were collected in 0.25% trypsin and washed three times with cold PBS. The apoptosis of chordoma cells was evaluated by Annexin V-PE/PI apoptosis detection kit (BD Biosciences, USA) by flow cytometry.

### Western blotting analysis

Chordoma cells or tumor tissue were collected and lysed in radioimmune precipitation assay buffer (Beyotime Biotechnology, China) containing a complete protease inhibitor cocktail (Roche, Germany), and the concentrations of protein were determined by Pierce BAC Protein Assay Kit (Thermo Fisher Scientific, USA). A certain amount of protein was mixed with SDS-polyacrylamide gel electrophoresis (PAGE) loading buffer (Beyotime Biotechnology, China), boiled for 15 min, and subjected to SDS-PAGE followed by transferring to polyvinylidene difluoride (PVDF) membranes (Merck Millipore, Germany). Blots were probed with primary antibodies at 4 °C overnight, including the following: anti-KRT8 antibody (Abcam, #ab53280, 1:1000), anti-LC3B (Abcam, # ab48394, 1:500), anti-SQSTM1 (Cell Signaling Technology, #5114, 1:1000), anti-PERK (Cell Signaling Technology, #3192, 1:1000), anti-p-PERK (Invitrogen, #PA5-40294, 1:1000), anti-eIF2α (Cell Signaling Technology, #5324, 1:1000), anti-p-eIF2α (Cell Signaling Technology, #3398, 1:500), anti-Caspase 4 (Cell Signaling Technology, #4450, 1:1000), anti-CHOP (Cell Signaling Technology, #7351, 1:1000), anti-XBP1s (Cell Signaling Technology, #40435, 1:500), anti-cleaved PARP (Santa Cruz Biotechnology, #56196, 1:500), anti-BiP (Santa Cruz Biotechnology, #13539, 1:1000), and anti-glyceraldehyde 3-phosphate dehydrogenase (GAPDH)f (Cell Signaling Technology, #2118, 1:1000). Then, incubate the membrane with horseradish peroxidase (HRP)-linked goat anti-rabbit IgG or horse anti-mouse IgG secondary antibody (Cell Signaling Technology, #7074 or #7076, Cell Signaling Technology) for 2 h. Each step was followed by three washings in TBS (Tris-Buffered Saline) containing 0.1% Tween-20 for 15 min. Finally, the PVDF membranes were detected using Immobilon Western Chemiluminescent HRP Substrate (#WBKLS0100, Millipore Corporation, Germany) and were observed under Amersham Imager 600 (General Electric, USA).

### Quantitative qRT‑PCR analysis

Total RNA of chordoma cells were collected using MiniBEST Universal RNA Extraction Kit (TaKaRa, Japan) according to the manufacturer’s instructions. Reverse transcription was performed with PrimeScript RT Master Mix (TaKaRa, Japan). Synthesized cDNA was then subjected to quantitative PCR analysis using TB Green Premix Ex Taq II (TaKaRa, Japan). The reactions were performed with CFX96 (Bio-Rad, USA). Gene expression levels were reported as relative fold change, with GAPDH as an internal control. Primers sequences used for qRT-PCR were shown in Supplementary Table 2.

### Immunofluorescence staining

Chordoma cells were fixed in freshly prepared 4% paraformaldehyde for 30 min and permeabilized by 0.1% Triton X-100 (MP Biomedicals, USA) for 30 min. Then the cells were and blocked by 0.8% bovine serum albumin in PBS for 1 h, incubated with primary antibody anti:LC3B (Abcam, #ab48394, 1:50), anti-CHOP (Cell Signaling Technology, #7351, 1:50), anti-BiP (Santa Cruz Biotechnology, #13539, 1:50), and anti-KRT8 (Abcam, #ab53280, 1:50) at 4 °C overnight. Then cells were incubated with fluorescein isothiocyanate (FITC)-conjugated Affinipure Goat Anti-Mouse IgG(H + L) (#SA00003-1, Proteintech, China) or FITC-conjugated Affinipure Goat Anti-Rabbit IgG(H + L) (#SA00003-2, Proteintech, China) at room temperature for 2 h. Finally, the cells were incubated with 4′,6-diamidino-2-phenylindole (C1006, Beyotime Biotechnology, China) for 10 min or rhodamine phalloidin (#PHDR1, Cytoskeleton, Inc, USA) for 30 min. Each step was followed by washing with PBS three times for 5 min each. Then the cells or sections were analyzed under a fluorescence microscope (BX53, OLYMPUS, Japan). For tumor sections, antigen retrieval was performed with citrate buffer (pH = 6.0).

### RT-PCR analysis

To evaluate relative splicing level of XBP1 mRNA, RT-PCR analysis was performed using SanTaq PCR Master Mix (Sangon, China). PCR products were analyzed on a 3.5% agarose gel. Primers sequences used for qRT-PCR were shown in Supplementary Table 3.

### Mice xenograft model and siRNA transfection in vivo

All animal experiments were approved and followed the guidelines issued by the Animal Experiment Administration Committee of the Fourth Military Medical University. Twelve 5-week-old male NOD/SCID mice were maintained under specific pathogen-free conditions. CM319 chordoma cells were collected and washed three times with cold PBS, then 100 μl of CM319 cells at a density of 1 × 10^7^ were injected subcutaneously into the NOD/SCID mice. Tumor volumes were examined every other day and the volume of the tumor (V) was approximately calculated as *V* = (length × width)^2^/2 (mm^3^). When the mean tumor volume reached 500 mm^3^ (15 days after the injection of CM319 cells), the NOD/SCID mice were randomly assigned (using a random number table) to four groups: three mice for each group—control group (Con), siKRT8 group (siKRT8), Doxo-treated group (Doxo, 2 mg/kg), and siKRT8 and Doxo-treated group (si + Doxo 2 mg/kg). Doxo were intraperitoneally administered every day for 14 days and siRNA was transfected three times weekly^59,60^. The chemically modified stable^TM^ siRNA of human KRT8 (siKRT8) for in vivo studies was manufactured by Genepharma (Suzhou, China). The stable^TM^ siRNA (20 μg) was dissolved in Optic-MEM medium (Thermo Fisher Scientific, USA, #31985062) and was injected to the tumor. After 30 days from the injection of the CM319 cells, the mice were killed and xenografts were collected for weighing and histology analysis. The investigator who measured the volume and weight of the tumor was blinded to the group allocation. No animal was excluded from the analysis.

### Statistical analysis

Statistical analysis was performed with SPSS 22.0 and GraphPad Prism 7.0 software. The results were given as mean ± SD. No statistical methods were used to determine the sample size for in vitro and in vivo experiments. An *F*-test for equality of variances was performed to ensure the same variance of tested groups. The Shapiro-Wilk test or D’Agostino test was performed to determine whether the data follow a normal distribution. Differences between experimental groups were assessed using one-way analysis of variance followed by Dunnett’s or Tukey’s multiple comparisons test. A *p* < 0.05 was considered statistically significant.

## Supplementary information

Supplementary Figure 1

Supplementary Figure 2

Supplementary Table and Supplementary Fig Legend
