# Supplementary figures and images for: Knockdown of cytokeratin 8 overcomes chemoresistance of chordoma cells by aggravating endoplasmic reticulum stress through PERK/eIF2α arm of unfolded protein response and blocking autophagy
Source: Cell Death Dis. 2019 Nov 25;10(12):887. doi: 10.1038/s41419-019-2125-9 (PMC6877560; doi:10.1038/s41419-019-2125-9)

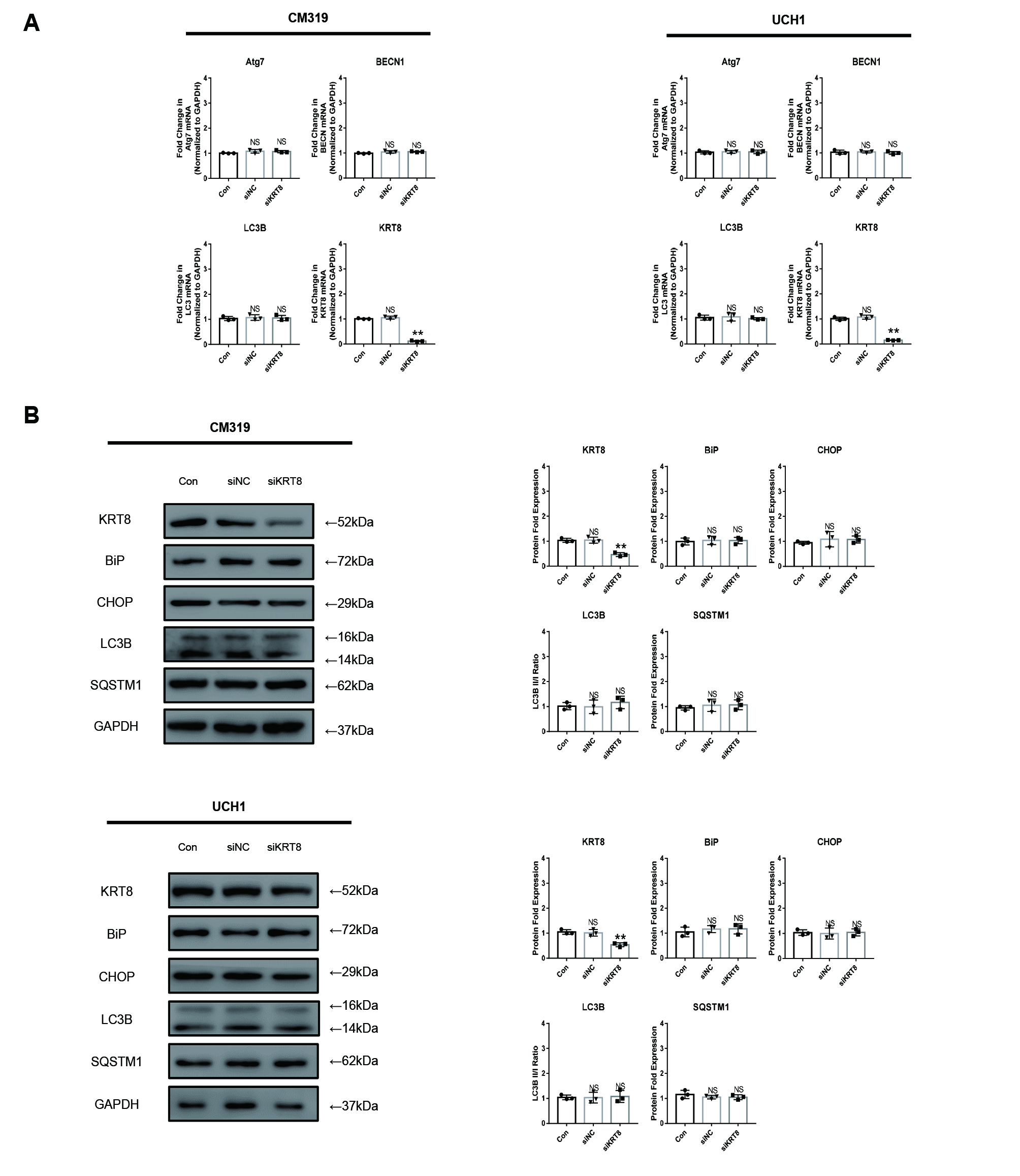

Supplement: Supplementary file 1 — Supplementary Figure 1 [file 41419_2019_2125_MOESM1_ESM.tif]

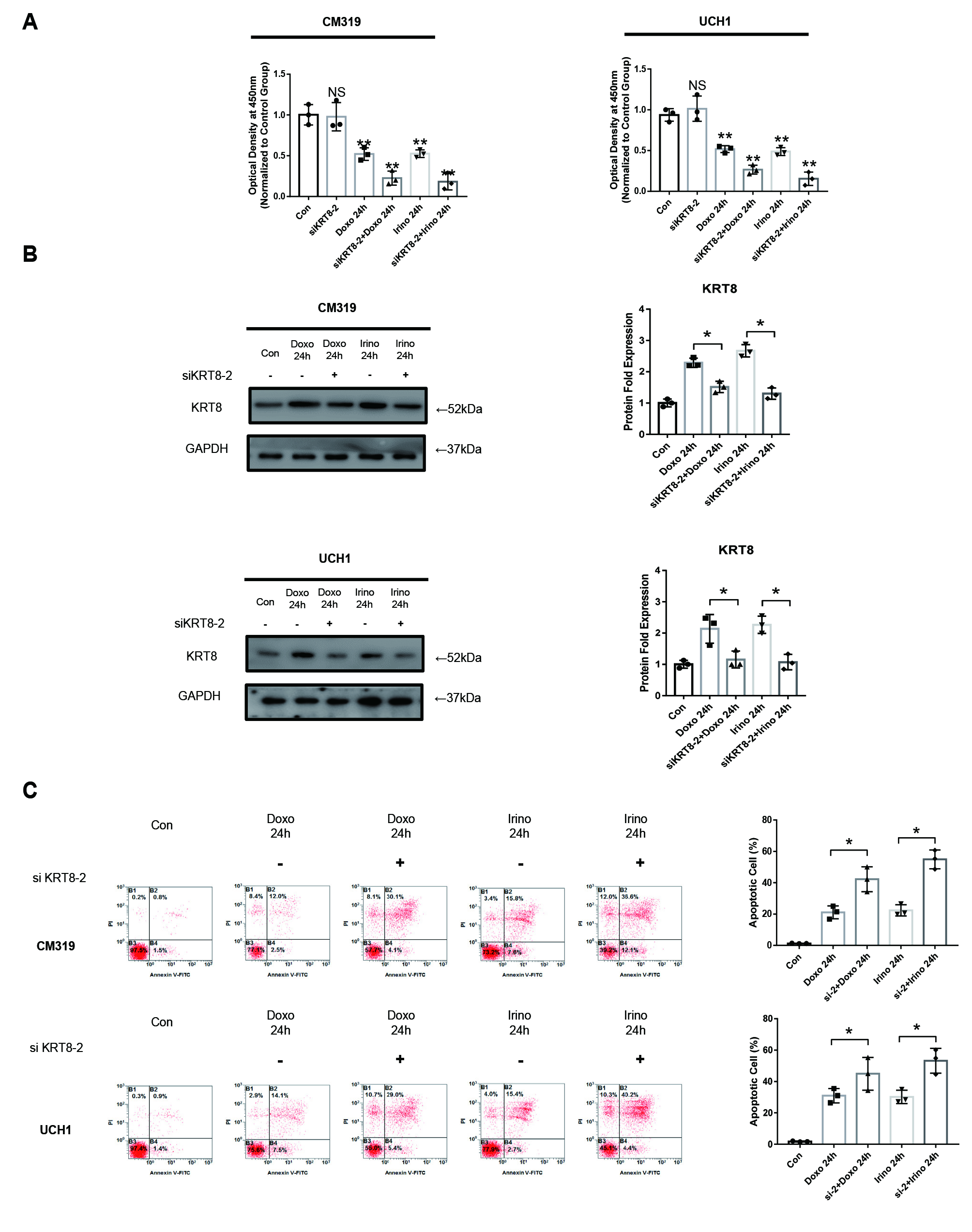

Supplement: Supplementary file 2 — Supplementary Figure 2 [file 41419_2019_2125_MOESM2_ESM.tif]
