# Supplementary material for: Knockdown of cytokeratin 8 overcomes chemoresistance of chordoma cells by aggravating endoplasmic reticulum stress through PERK/eIF2α arm of unfolded protein response and blocking autophagy
Source: Cell Death Dis. 2019 Nov 25;10(12):887. doi: 10.1038/s41419-019-2125-9 (PMC6877560; doi:10.1038/s41419-019-2125-9)
Supplement: Supplementary file 3 — Supplementary Table and Supplementary Fig Legend [file 41419_2019_2125_MOESM3_ESM.docx]

Supplementary Table 1. Sequences of siRNA

| Name | sense(5’-3’) | antisense(5’-3’) |
| --- | --- | --- |
| siKRT8 | GCAUCAGCUCCUCGAGCUUTT | AAGCUCGAGGAGCUGAUGCTT |
| siKRT8-2 | GGGAGGCAUCACCGCAGUUTT | AACUGCGGUGAUGCCUCCCTT |

Supplementary Table 2. Sequences of primers used for qRT-PCR.

| Gene (Homo) | Sequence(5′–3′) | |
| --- | --- | --- |
| Atg7 | Sense | CAGTTTGCCCCTTTTAGTAGTGC |
|  | Antisense | CCAGCCGATACTCGTTCAGC |
| BECN | Sense | CCATGCAGGTGAGCTTCGT |
|  | Antisense | GAATCTGCGAGAGACACCATC |
| LC3B | Sense | GATGTCCGACTTATTCGAGAGC |
|  | Antisense | TTGAGCTGTAAGCGCCTTCTA |
| KRT8 | Sense | CAGAAGTCCTACAAGGTGTCCA |
|  | Antisense | CTCTGGTTGACCGTAACTGCG |
| GAPDH | Sense | GGAGCGAGATCCCTCCAAAAT |
|  | Antisense | GGCTGTTGTCATACTTCTCATGG |

Supplementary Table 3. Sequences of primers used for RT-PCR.

| Gene (Homo) | Sequence(5′–3′) | |
| --- | --- | --- |
| XBP1 | Sense | CCTGGTTGCTGAAGAGGAGG |
|  | Antisense | CCATGGGGAGATGTTCTGGAG |
| β-actin | Sense | GGGTCAGAAGGATTCCTATG |
|  | Antisense | GGTCTCAAACATGATCTGGG |

Supplementary Figure Legend

FigS1. Knockdown of KRT8 using siRNA *in vitro*.

Chordoma cell line CM319 and UCH1 were transfected with siKRT8.

(A) Atg7, BECN1, LC3B, KRT8 mRNA level was determined by qRT-PCR.

(B) Western blot analysis and quantification of KRT8, BiP, CHOP, LC3B, SQSTM1 (Normalized to GAPDH expression).

(n=3; *: p＜0.05 versus control group; **: p＜0.01 versus control group, NS: not statistically significant versus control group; Con: control group. For all the above-mentioned statistical analyses, significance was determined by One-way ANOVA followed by Dunnett's multiple comparisons test, and results were shown as mean ± S.D.)

FigS2. siKRT8-2 chemosensitizing chordoma cells *in vitro*.

Chordoma cell line CM319 and UCH1 were transfected with siKRT8-2 followed by treatment of doxorubicin (0.5μM) or irinotecan (50μM) for 24h.

(A) Cell viability of chordoma cells were determined by CCK8 assay.

(B) Western blot analysis and quantification of KRT8 protein expression (Normalized to GAPDH expression).

(C) Apoptosis of chordoma cells was determined by Annexin V-PE/PI staining measured by flow cytometry.

(n=3; *: p＜0.05 versus indicated group; **: p＜0.01 versus indicated group, NS: not statistically significant versus indicated group; Con: control group; Doxo: doxorubicin treated group; Irino: irinotecan treated group. For all the above-mentioned statistical analyses, significance was determined by One-way ANOVA followed by Tukey’s multiple comparisons test , and results were shown as mean ± S.D.)
